# Prediction of 10-year Overall Survival in Patients with Operable Cervical Cancer using a Probabilistic Neural Network

**DOI:** 10.7150/jca.33945

**Published:** 2019-07-10

**Authors:** Bogdan Obrzut, Maciej Kusy, Andrzej Semczuk, Marzanna Obrzut, Jacek Kluska

**Affiliations:** 1 Medical Faculty, University of Rzeszow, Rejtana str. 16C, 35-959 Rzeszow, Poland; 2 Department of Obstetrics and Gynecology, Provincial Clinical Hospital No. 2, Lwowska str. 60, 35-301 Rzeszow, Poland; 3 Faculty of Electrical and Computer Engineering, Rzeszow University of Technology, Powstanców Warszawy avenue 12, 35-959 Rzeszow, Poland; 4 II ND Department of Gynecology, Lublin Medical University, Jaczewski str. 8, 20-954, Lublin, Poland

**Keywords:** cervical cancer, 10-year overall survival, probabilistic neural network, survival prediction

## Abstract

**Background**: Toward the goal of predicting individual long-term cancer survival to guide treatment decisions, this study evaluated the ability of a probabilistic neural network (PNN), an established model used for decision-making in research and clinical settings, to predict the 10-year overall survival in patients with cervical cancer who underwent primary surgical treatment.

**Patients and Method**: The input dataset was derived from 102 patients with cervical cancer FIGO stage IA2-IIB treated by radical hysterectomy. We identified 4 demographic parameters, 13 tumor-related parameters, and 6 selected perioperative variables for each patient and performed computer simulations with DTREG software. The predictive ability of the model was determined on the basis of its error, sensitivity, and specificity, as well as area under the receiver operating characteristic curve. The results of the PNN predictive model were compared with those of logistic regression analysis and a single decision tree as reference models.

**Results**: The PNN model had very high predictive ability, with a sensitivity of 0.949, a specificity of 0.679, and an error rate of 12.5%. The PNN's area under the receiver operating characteristic curve was high, 0.809, a value greater than those for both logistic regression analysis and the single decision tree.

**Conclusion**: The PNN model effectively and reliably predicted 10-year overall survival in women with operable cervical cancer, and may therefore serve as a tool for decision-making process in cancer treatment.

## Introduction

Cervical cancer is the fourth most common malignancy among women worldwide 1. In Poland, cervical cancer accounted for 3.6% of all newly registered carcinomas in women in 2015, making it the seventh most frequent female genital tract malignancy. Moreover, cervical carcinoma is the eighth leading cause of cancer-related death among Polish women 2.

Due to improved living conditions, growing health awareness, and the development of diagnostic, screening, and therapeutic methods over the last several decades, as well as the increasing life expectancy of the general population, cancer patients may also be surviving longer than before. Longer patient survival and delayed cancer deaths have been clearly confirmed in recently published data on many hematologic malignancies and solid tumors in humans, including cervical cancer 3-5. Given these observations, long-term survival rates (10, 15, or even 20 years) are becoming increasingly important outcome measures of cancer and are routinely reported by cancer registries worldwide 3,6,7. The established estimates of long-term survival are useful for both clinical-practice and public-health purposes. They also enable estimation of general outcomes. They cannot, however, predict survival in an individual patient, a capability that might be very useful for planning individual follow-up.

A probabilistic neural network (PNN) - an advanced computer program that can analyze datasets and uncover complex relationships within them undetectable through logistic regression (LR) analysis - has been used to predict the 5-year overall survival of cervical cancer patients 8. To our knowledge, however, there are no tools currently available for predicting 10-year survival in cervical cancer patients.

To achieve the goal of individualized prediction, in this study we sought to develop a universal PNN model for predicting 10-year overall survival in individual women with cervical cancer utilizing demographic characteristics, tumor-related parameters, and select perioperative data.

## Patients and Methods

The study was approved by the Bioethics Committee of the Regional Medical Chamber, Rzeszow, Poland (reg. no. 3/98; 20/02/1998). Written informed consent was obtained from all the patients for their data to be used for investigation.

The study enrolled 117 women with cervical cancer classified as International Federation of Gynecology and Obstetrics (FIGO) stage IA2-IIB, who were operated on between 1998 and 2001 at the Department of Obstetrics and Gynecology of the Rzeszow State Hospital in Poland.

All of the patients underwent radical abdominal hysterectomy Piver class III and pelvic lymphadenectomy. Perioperative complications were prospectively recorded according to the classification proposed by Chassagne *et al.* 9.

After the postoperative recovery period, some patients received adjuvant radiotherapy. The inclusion criteria were as follows: lymph node metastases, lymph-vascular space invasion (LVSI), neoplastic tissue within the surgical incision, or non-squamous types of cervical cancer. Radiotherapy was administered as follows: teletherapy (50 Gy to the area of the pelvis minor in 25 fractions of 2 Gy; BOX technique) and brachytherapy (two fractions of low-dose rate; total dose, 30 Gy). No patient from the study group received chemotherapy, since it was not routinely applied during the study period.

Follow-up was conducted every month during the first year after surgery, every 3 months during the second year, twice annually for 3-5 years, and then once per year. The 10-year follow-up data from all subjects were used to validate the examined computational intelligence models designed to predict death within 120 months.

Data available at the time of discharge, derived from histopathologic examination of the surgical specimens and data obtained during the follow-up were collected. In total, 23 variables were identified and sub-divided into 3 groups. The first group of variables comprised four demographic characteristics: age, body mass index (BMI), hormonal status, and the presence of concomitant diseases. The second group comprised 13 tumor-related parameters: FIGO stage, histologic type, histologic grade, tumor size (≤ 4 cm or > 4 cm), lymph node status, number of lymph nodes dissected, number of positive lymph nodes, lymph node ratio (ratio of positive to totally removed lymph nodes), lymph-vascular space invasion (LVSI), surgical-margin status, parametrial involvement, deep stromal invasion (outer one-third of the cervical stroma), and postoperative radiotherapy (Table 1). The third group included six selected perioperative variables: surgery time, blood loss, presence of intraoperative complications, presence of postoperative complications, types of complications, and length of hospital stay (Table 2).

All the above variables were used in computer simulations applying the PNN developed by Specht 10, which is available in DTREG software 11. The error, sensitivity, specificity, and area under the receiver operating characteristic curve (AUROC) were used to determine the predictive ability of the applied model. The above parameters were obtained through a 10-fold cross-validation procedure 12. The simulations were performed 20 times by assuming a random selection of training and test subsets. The results were averaged and the standard deviations calculated. As a reference model, we used LR analysis and a single decision tree (SDT), both of which are widely applied in medical research 13-17.

### Software

PNN, SDT, and LR survival analyses were performed with DTREG software (version 10.7.18, Phillip H. Sherrod, www.dtreg.com, USA) and Matlab (version R2018b, MathWorks, Inc., www.mathworks.com, USA).

### Statistical analysis

The AUROC values for the PNN and reference models were compared with two-tailed pairwise *t*-tests. A *p* value of less than 0.05 was considered statistically significant. All statistical analyses were performed in MathWorks' Matlab R2018b software.

## Results

A total of 117 patients qualified for a radical Piver III hysterectomy and pelvic lymphadenectomy. Of these 117, 15 were excluded from the analysis (3 patients whose final histopathologic findings revealed endometrial cancer with cervical extension; 4 patients who continued postoperative treatment and follow-up at another institution; 3 patients who refused to participate in the study protocol; and 5 patients who were lost during the follow-up). The remaining 102 patients were considered eligible and were enrolled in the study.

Among the 102 included patients (mean patient age 48 years, range 29-73; mean ± SD BMI [kg/m^2^] 25.9 ±4.9), hormonal status was premenopausal in 71 and postmenopausal in 31. Concomitant diseases were found in 33: arterial hypertension in 21, diabetes mellitus in 3, ischemic heart disease in 6, and other diseases in 3.

The prevailing histologic cervical tumor type was squamous-cell carcinoma (90%). The clinicopathologic parameters are reported in Table 1.

The perioperative parameters of the study group are shown in Table 2. Perioperative complications affected 46.1% of the patients. Most adverse events were mild or moderate complications that did not threaten patient health or life. Severe perioperative complications (pulmonary embolism, bleeding from the vena cava inferior, rupture of duodenal ulcer or genito-urinary fistulas) occurred in seven patients. Additional perioperative data are shown in Table 2.

The mean follow-up period was 95.5 months (range 6-120 months). During the follow-up, recurrence was identified in 28 patients (27.5%). Pelvic recurrence was detected in 15 patients and the remaining 13 subjects were diagnosed with distant metastases. During the final observations, 74 patients were alive, and 28 had died from cancer-related causes. The overall 10-year survival was 72.5%.

**Figure 1:**
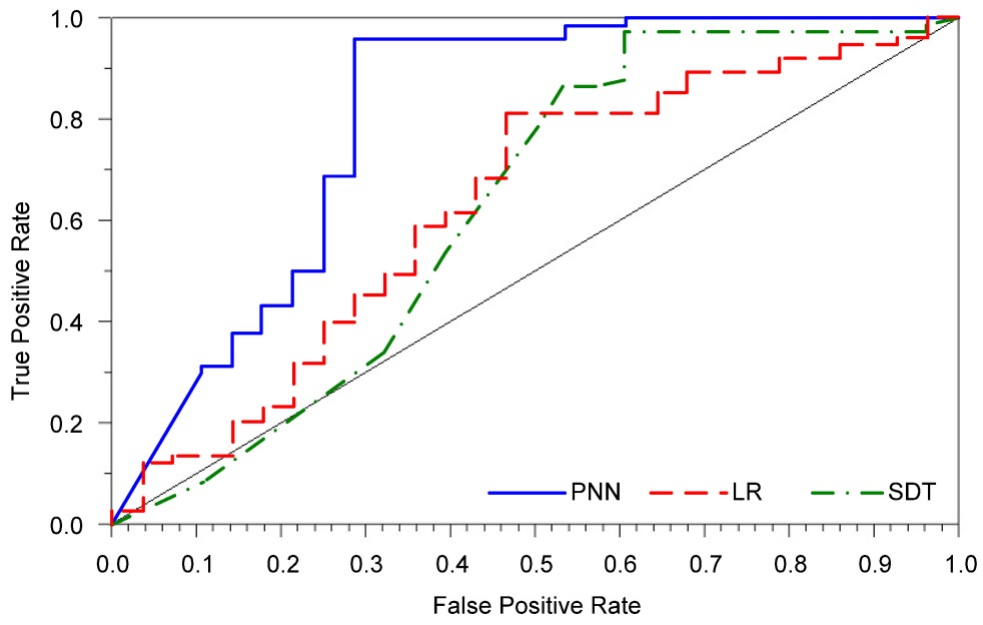
Receiver operating characteristic curves for all prediction models.

The applied PNN model was used to predict the 10-year overall survival in cervical cancer patients treated with radical hysterectomy. The error, sensitivity, and specificity for the PNN were markedly better than those obtained by the LR model (Table 3). Although the SDT model had the highest sensitivity, its prediction error was higher than that of the PNN. The AUROC for the PNN was also substantially greater than that for both the LR and SDT models (Figure 1).

Of the 28 cases of patient death, 10 were incorrectly predicted to survive (Table 4). On the other hand, only four cases were misclassified among the patients who survived during follow-up.

## Discussion

Probabilistic neural networks are established tools for classifying medical data 18, 19, and were recently used for predicting complications as well as 5-year overall survival in cervical cancer patients treated with radical hysterectomy 8, 20, 21. A PNN model for predicting long-term overall survival to aid in treatment decision-making, however, was lacking. A search of the PubMed^®^ database revealed that there are no previously published reports of the application of a PNN to predict the effects of radical hysterectomy on 10-year overall survival in cervical cancer patients. This is also the first investigation of prognostication of 10-year survival in these patients.

Operationally, the most important advantage of PNN is that training is easy and instantaneous 10. As additional patterns (e.g., records involving patient data) are observed and stored in the network, the generalization property of this network as the most important feature of each classification algorithm, will improve. Other advantages of the PNN are as follows: the shapes of the decision surfaces can be made as complex as necessary or as simple as desired by choosing the appropriate value of the smoothing parameter; the decision surfaces can approach the Bayes optimum; erroneous samples are tolerated; sparse samples are adequate for network performance; for time-varying statistics, old patterns can be overwritten with new patterns; and unlike many artificial neural networks, PNN operates completely in parallel.

Our simulations were performed using mostly prognostic factors that are well-established in uterine cervical carcinoma. The evidence for the included variables is discussed below. Clinical disease stage was included because it is a well-established critical variable 22, 23. The FIGO stage was included because it was recently reported to be an independent prognostic factor for 10-year overall survival in women with cervical cancer 24, 25. Although previous studies failed to confirm that tumor size is an independent prognostic factor for the 10-year survival rate 24, 25, we included tumor size because it correlates with outcome for patients with cervical carcinoma 26-28. Additional established prognostic factors included in the study were parametrial infiltration and depth of stromal invasion of the cervix 25,29-31. We also included histologic subtype, although no conclusive evidence regarding its effects on patient outcomes in cervical cancer has been presented 32. Some studies found that 5-year survival rates do not differ significantly between squamous cell carcinoma and adenocarcinoma 33, 34, whereas others report that the 5-year survival rates are unfavorable in women with cervical adenocarcinoma 35, 36. In our previous study with long-term follow-up 24, we identified a tendency toward higher 10-year disease-free survival and 10-year overall survival rates for patients with squamous cell carcinoma than for patients with adenocarcinoma. In agreement, Suprasert *et al.* 25 reported that histologic type of cervical cancer other than squamous cell-carcinoma was an independent unfavorable prognostic factor during a 10-year follow-up.

Despite the controversy surrounding histologic grade as a prognostic factor for patients with cervical cancer, we included histologic grade in the present study. Several findings indicate an unfavorable 5-year outcome for patients with poorly differentiated squamous cell cervical carcinoma 37, but other studies have not confirmed these observations 38, 39. We previously reported a tendency toward lower 10-year survival rates for patients with poorly (G3) differentiated cervical carcinoma compared with patients with G1 and G2 tumors 24.

Lymph node involvement, another parameter included in the present study, is closely related to 5-year survival in cervical cancer patients 24, 40-43. The most recently reported data indicate that 10-year disease-free and overall survival are significantly lower for patients with positive pelvic lymph nodes than for patients without lymph node metastases 24, 25.

LVSI is associated with an unfavorable outcome in cervical cancer patients. Although the correlation between LVSI and lymph node involvement is high, some researchers consider LVSI an independent factor for predicting 5-year survival 44, 45. We recently reported in a long-term follow-up study that 10-year overall survival rates are significantly higher in patients without LVSI than in those with LVSI 24. Interestingly, in that study, we also found that the 10-year disease-free survival rates tended to be higher for patients without LVSI 24.

We included patient age, although the literature includes contradictory data regarding the effect of patient age on 5-year overall survival in patients with cervical cancer. Several studies report no differences in survival rates among different age groups 46, 47, whereas others suggest that younger patients have a significantly poorer prognosis 48,49. In our previous long-term follow-up study, we found no association between patient age and 10-year overall survival 24.

A positive surgical margin was included in the present study because it is a well-established important risk factor for cervical cancer recurrence. The effect of positive surgical margins on either 5-year or 10-year overall survival in cervical cancer patients has not been supported by multivariate analyses 24, 37, 50.

Available data do not support a direct effect of surgery duration or length of hospitalization on patient survival. Nevertheless, we included various perioperative variables as related to both the course of the operation and convalescence, and thus may also relate to complications. Similarly, perioperative complications themselves do not affect the natural history of carcinoma, but the treatment of perioperative complications may delay administration of adjuvant therapy, consequently negatively affecting patient prognosis. Comorbidity as an indicator of general patient health, may also limit the administration of adjuvant therapy, which might worsen the prognosis 51.

In our PNN model, we applied a total of 23 variables (see above: demographic characteristics, tumor-related parameters, and select perioperative data) because a larger number of factors improves the accuracy of artificial neural networks 52, and even variables without a statistically significant effect may still have some effect on survival 53. Our model could predict 10-year overall survival in cervical cancer patients with an error of 12.5%. The probabilistic neural network had high sensitivity but relatively low specificity, possibly as a result of class imbalance, i.e., the study population had a very low number of deaths 8.

Unfortunately, we cannot compare our findings with results from other studies due to the small number of relevant publications. Only a few studies in the current literature predict survival of cervical cancer patients, and they reported only 5-year overall survival. For example, Ochi *et al.* 54 used artificial neural networks to predict the effects of radiotherapy on survival of cervical cancer patients. Polterauer *et al.* 53 developed a nomogram for patients with uterine cervical carcinoma, FIGO stages IB-IV, whereas Zhou *et al.* 55 established a nomogram predicting the effects of surgical treatment on survival in patients with stage IA-IIB cervical cancer. All three models predicted 5-year overall survival with an AUROC value of 0.71-0.778. Our recently published results predicting 5-year overall survival in cervical cancer patients with FIGO stage IA2-IIB cancer using computational intelligence methods outperformed all the aforementioned studies (AUROC value for PNN model of 0.818) 8. These findings suggest that the present study, based on similar methodology, provides satisfactory and credible findings.

This study has some limitations. First, clinical data from patients operated on between 1998 and 2001 were used for the simulations. Since then, the treatment standards for cervical cancer substantially changed. In this context, it may seem controversial that we included patients with FIGO stage IIB cervical cancer. The current recommendations suggest that cervical cancer FIGO stage IIB should be treated with definitive chemo-radiation 56-58. The latest NCCN-Guidelines (version 1.2019), however, state that “in some countries, select cases of stage IIB may be treated with upfront radical hysterectomy or neoadjuvant chemotherapy followed by radical hysterectomy” 56. Surgical treatment of cervical cancer FIGO stage IIB is common in China, Korea, and Japan 59-62. The German Cancer Society and German Society of Gynaecology and Obstetrics also recently recommended that FIGO stage IIB cervical cancer can be treated by radical hysterectomy with adjuvant radiotherapy 63. Poland is among those countries with a longstanding surgical tradition 24, 43. We emphasize that in our series of radical hysterectomy, selected cases of FIGO stage IIB cervical cancer were qualified by early parametrial infiltration (proximal portion of the lateral parametria) and associated pathologies of the female genital tract, including uterine fibroids, adnexal tumors, or massive ventral hernia in a post-laparotomy scar, as well as comorbid psychiatric disorders precluding radiotherapy. The survival outcomes for patients in the present study were comparable to those previously reported 25, 59, 64, 65, thus indirectly confirming our decisions. Second, concurrent chemo-radiotherapy has been introduced on a large scale. In this context, our results refer only to a select group of patients. Finally, the number of patients was limited to allow us to analyze in detail the effects of histologic type on survival in patients with non-squamous cell carcinoma. This category also included patients with adenocarcinomas and glandular squamous, microcellular, and undifferentiated cervical carcinomas.

The current study has several strengths. The main strength is the study design. As previously mentioned, on the basis of a PubMed^®^ database search, this is the first report applying PNNs for predicting 10-year overall survival in cervical cancer patients treated with radical hysterectomy. Moreover, the entire series of patients was treated in a single institution in which the same principles of diagnostic and surgical procedures were carefully matched. This aspect markedly enhances the strength of our study.

## Conclusion

The PNN model effectively and reliably predicted 10-year overall survival in women with operable cervical cancer, and may therefore serve as a tool for decision-making processes in cancer treatment.

## Figures and Tables

**Table 1:** Clinicopathologic data of the study group.

Clinical stage n (%)	IA2	15 (15)	
	IB1	51 (50)	
	IB2	8 (8)	
	IIA	7 (7)	
	IIB	21 (20)	
Histologic type n (%)	Squamous	91 (90)	
	Non-squamous	11 (10)	
Histologic grade n (%)	G1	19 (19)	
	G2	62 (61)	
	G3	21 (20)	
Tumor size n (%)	≤ 4 cm	69 (68)	
	> 4 cm	33 (32)	
Mean number of removed lymph nodes (range)		13.8 (1-40)	
Lymph node status n (%)	Negative	77 (76)	
	Positive	25 (24)	
Mean number of positive lymph nodes (range)		0.5 (1-9)	
Lymph node ratio (range)		0.068 (0-1)	
LVSI n (%)	Absent	83 (82)	
	Present	19 (18)	
Deep stromal invasion n (%)	Absent	66 (65)	
	Present	36 (35)	
Parametrium infiltration n (%)	Absent	78 (77)	
	Present	24 (23)	
Surgical-margin status n (%)	Negative	89 (88)	
	Positive	13 (12)	
Postoperative radiotherapy n (%)	Yes	57 (56)	
	No	45 (44)	

**Table 2:** Perioperative parameters in the study group.

Mean surgery time (min, range)		194.7 (80-310)	
Mean blood lost (△Hb; g%; range)		3.8 (0.3-7.8)	
Intraoperative complications (n)		5	
Postoperative complications (n)		42	
Types of complications according to Chassagne *et al.* 9 n (%)	Mild	38 (81)	
	Moderate	2 (4)	
	Severe	7 (15)	
Mean hospital stay (days, range)		12.7 (5-49)	

**Table 3:** Error, sensitivity, specificity, and AUROC for PNN and the reference predicting models after a 10-fold cross-validation procedure. The results are shown as means for 20 simulations, with standard deviations in parentheses.

	Error	Sensitivity	Specificity	AUROC
PNN	0.125	0.949	0.679	0.809
	(0.024)	(0.010)	(0.064)	(0.026)
LR	0.300	0.816	0.393	0.622
	(0.008)	(0.018)	(0.032)	(0.012)
SDT	0.196	0.965	0.379	0.624
	(0.018)	(0.011)	(0.036)	(0.013)

**Table 4:** Confusion matrix for the probabilistic neural network.

			Predicted outcome	
Actual outcome			Died	Survived
Died			18	10
Survived			4	70

## Abbreviations

AUROC: Area Under Receiver Operating Characteristic curve
BMI: Body Mass Index
FIGO: The International Federation of Gynecology and Obstetrics
LR: Logistic Regression
LVSI: Lymph-Vascular Space Invasion
PNN: Probabilistic Neural Network
SDT: Single Decision Tree
